# Carcinoid Heart Syndrome With Metastatic Low-Grade Neuroendocrine Tumor of the Liver: A Rare Case

**DOI:** 10.7759/cureus.59885

**Published:** 2024-05-08

**Authors:** Islam A Obeidat, Rabia Latif, Khurram Arshad, Athar Ghannam, Farman Ali, Rebecca Pratiti

**Affiliations:** 1 Internal Medicine, McLaren Hospital, Flint, USA; 2 Internal Medicine, Corewell Health East Dearborn, Dearborn, USA; 3 Family Medicine, McLaren Hospital, Flint, USA; 4 Medicine, St. John Hospital and Medical Center, Detroit, USA

**Keywords:** carcinoid tumors, report of a rare case, neuroendocrine tumors, tricupsid regurgitation, carcinoid heart disease

## Abstract

We present a rare and complex case of a 76-year-old male patient with a history of low-grade neuroendocrine tumor (NET) of the small intestine, status post resection, who presented with recurrence of the tumor in the liver and subsequent carcinoid heart syndrome (CHS). The recurrent liver tumor caused severe tricuspid regurgitation and CHS, highlighting the rare association between NETs and CHS, particularly in the elderly population. This case underscores the importance of multidisciplinary care and close monitoring for patients with recurrent NETs and potential cardiac complications.

## Introduction

Carcinoid tumors, emerging from neural crest cells, constitute a rare subset of neuroendocrine tumors (NETs), with an incidence rate ranging from 2.5 to 5.0 cases per 100,000 individuals annually [1]. Characterized by their potential for metastasis, particularly to the liver, these tumors pose a clinical challenge due to the release of excessive vasoactive amines into the systemic circulation [2]. Among the complications associated with carcinoid syndrome, carcinoid heart disease stands out prominently, encompassing a spectrum of cardiac manifestations, including tricuspid regurgitation. In this case report, we delve into the clinical journey of a patient diagnosed with a carcinoid tumor who subsequently developed carcinoid heart disease, culminating in heart failure secondary to severe tricuspid regurgitation [3]. Through this case, we aim to elucidate the intricate interplay between carcinoid tumors and cardiac pathology, shedding light on diagnostic and therapeutic considerations in managing this complex condition.

## Case presentation

We present a 76-year-old male with a complex medical history, including prior treatment for prostate cancer, duodenal ampullary adenoma resection, type 2 diabetes, dyslipidemia, and congestive heart failure of unknown ejection fraction. He underwent successful resection for a well-differentiated NET (stage T2N1) in the small intestine in 2015. The patient did not require radiation or chemotherapy, as this was a limited-stage disease. The patient consistently followed up with oncology until 2018, after which he stopped following up for unknown reasons. In 2023, the patient presented to his primary care physician with chronic diarrhea and abdominal pain, which prompted a workup that included an abdominal CT scan. The abdominal CT demonstrated an exophytic liver mass. The patient was referred to oncology for further evaluation.

During his oncology appointment, the patient experienced sudden substernal chest pain with an intensity of 7/10, lasting 10 minutes, and resolving spontaneously. Accompanied by shortness of breath, he denied palpitations, dizziness, dyspnea on exertion, orthopnea, or recent fluid intake changes. There was no ongoing nausea or vomiting, and his chronic diarrhea, which had lasted for four weeks, improved with symptomatic treatment. The patient was then sent to the emergency department for further evaluation of his chest pain. Upon arrival to the emergency room, vital signs included a blood pressure of 89/59 mmHg, a pulse rate of 66 beats per minute, temperature of 97.7°F, respiratory rate of 18 breaths per minute, height of 160 cm, weight 66.9 kg, pulse oximetry reading of 99%, and BMI of 25.10 kg/m². The patient, alert with a Glasgow coma score of 15/15, exhibited S1 and S2 with a systolic murmur at the lower middle sternal border and decreased air entry on the right lower lung field with bilateral crackles on chest auscultation. There was 2+ lower extremity pitting edema up to mid-thighs bilaterally. The abdominal exam was significant for moderate-to-large ascites. Pertinent lab investigations are summarized in Table 1 below.

**Table 1:** Pertinent lab investigations upon the patient's arrival at the hospital Hs-cTnT: high-sensitivity troponin T; NT-proBNP: B-type natriuretic peptide; ALP: alkaline phosphatase; WBC: white blood cells; AFP: alpha-fetoprotein; CEA: carcinoembryonic antigen; CA19-9: cancer antigen 19-9 Normal values are listed in between brackets.

Investigations	Results	Reference range
Hs-cTnT	27>28 ng/L	(0-19 ng/L)
NT-proBNP	1028 pg/ml	(0-450 pg/ml)
ALP	208 units/L	(41-126 units/L)
Total bilirubin	1.9 mg/dl	(0.3-1.2 mg/dl)
Serum serotonin	941 ng/ml	(56-244 ng/ml)
Serotonin blood	876 ng/ml	(56-244 ng/ml)
Creatinine	1.5 mg/dl	(0.6-1.5 mg/dl)
Calcium	9.3 mg/dl	(8.7-10.3 mg/dl)
Chloride	100 mmol/L	(96-109 mmol/L)
WBC	3.79 K/mcl	(4.5-10 K/mcl)
Hemoglobin	13.1gm/dl	(13-17 gm/dl)
Sodium	139 mmol/L	(135-145 mmol/L)
Potassium	3.9 mmol/L	(3.5-5.5 mmol/L)
Chromogranin\A	3006 ng/ml	(0-103 ng/ml)
AFP	<1.82 ng/ml	(0-7.9 ng/ml)
CEA	4.9 ng/ml	(0.0-34.9 units/ml)
CA19-9	2.1 units/ml	(0.0-34.9 units/ml)
Bicarbonate	24.5 mmol/L	(21.6-31.8 mmol/L)

**Figure 1:**
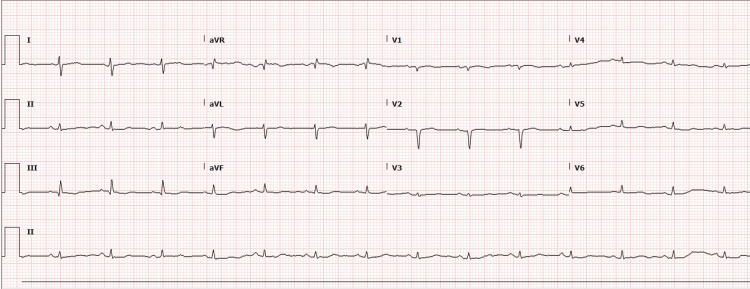
An EKG showed with no acute ischemic changes

**Figure 2:**
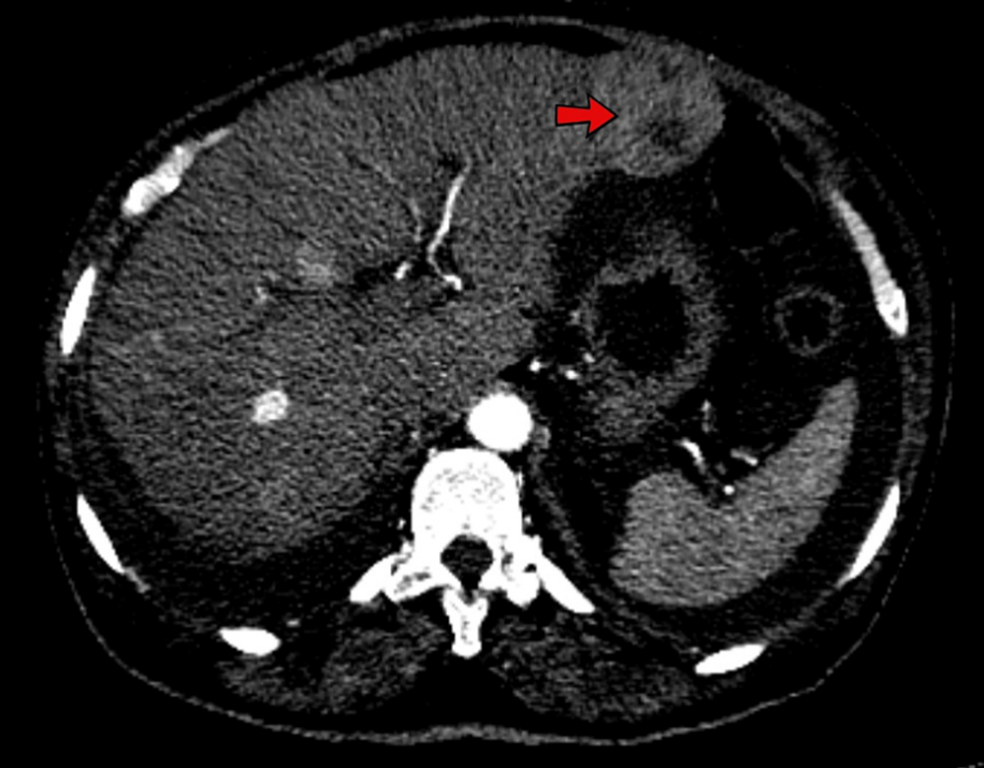
A CT scan of the chest, abdomen, and pelvis showed 4.4 x 5 cm of liver mass in the left lobe.

**Figure 3:**
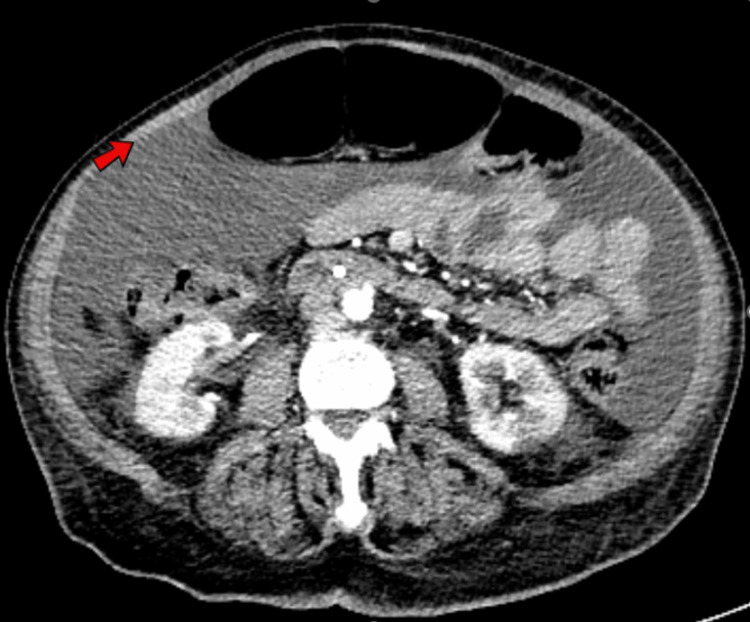
A CT scan of the chest, abdomen, and pelvis showed moderate ascites in the peritoneal cavities (red arrow) and borderline enlarged mesenteric and para-aortic lymph nodes.

**Figure 4:**
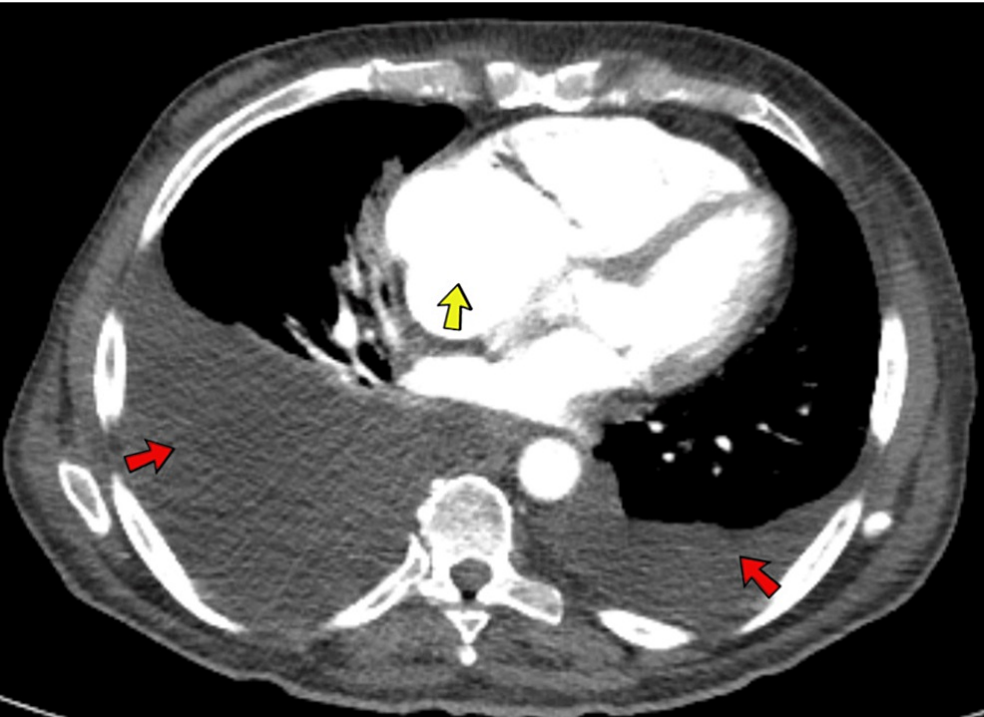
A CT scan of the chest, abdomen, and pelvis showed large right-sided pleural effusions and small to moderate-sized left-sided pleural effusions (red arrows). Increased right atrial size (yellow arrow) with a borderline enlarged heart.

**Figure 5:**
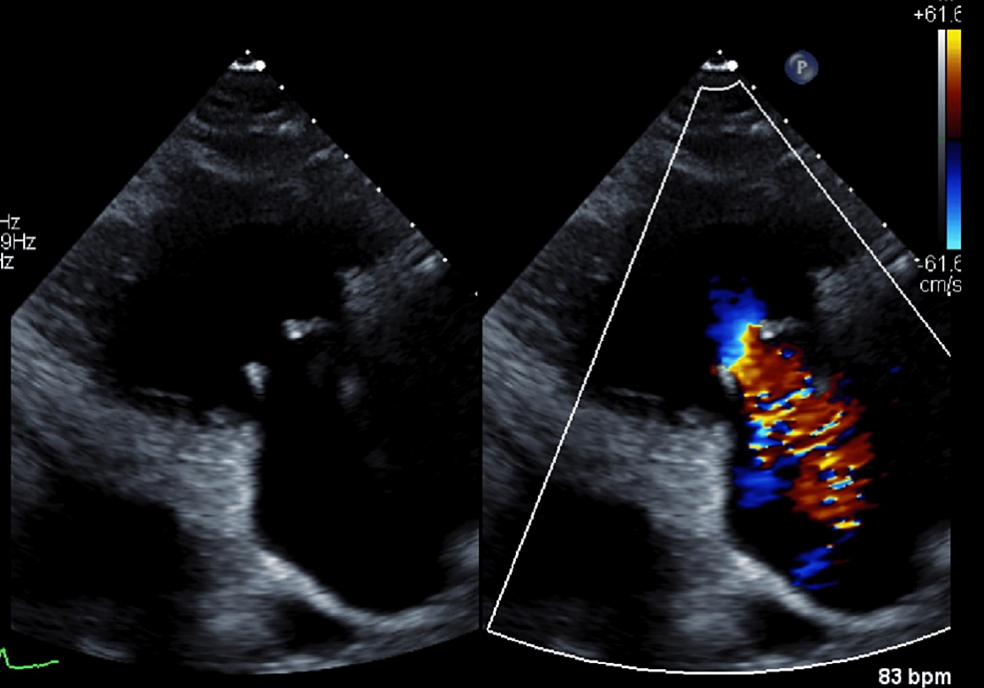
A color Doppler ultrasound of the tricuspid valve, with a jet of blood flowing downward (away from the transducer), indicated tricuspid regurgitation.

**Figure 6:**
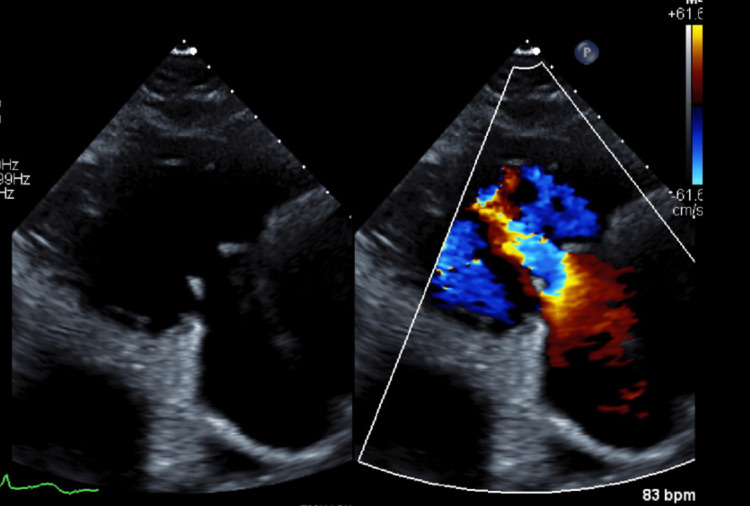
A color Doppler ultrasound of the tricuspid valve showed a jet of blood flowing downward (away from the transducer), indicating tricuspid regurgitation.

The EKG revealed a low-voltage QRS complex with no acute ST or T wave changes, as shown in Figure 1. A comprehensive CT scan of the chest, abdomen, and pelvis exposed a concerning 5 cm liver mass in the left lobe, suggesting malignancy. Imaging also indicated borderline enlarged mesenteric and para-aortic lymph nodes, moderate ascites, and bilateral pleural effusion (Figures 2-4). An echocardiogram showed a preserved ejection fraction of 55%-60%. However, there was marked enlargement of the right ventricle and right atrium, with mild pulmonary hypertension (right ventricular systolic pressure (RVSP) of 49.1 mmHg) and severe tricuspid regurgitation (Figures 5, 6). Given non-exertional chest pain, negative EKG changes with troponins in the normal range of chest pain were labeled as non-cardiac in nature.

The patient underwent paracentesis, which resulted in the removal of 2.4 liters of fluid. A right-sided ultrasound-guided thoracentesis extracted 1.3 liters of straw-colored fluid, confirmed as exudative through pleural fluid analysis (Table 2). A summary of the procedure and fluid analysis results are presented in Table 2.

**Table 2:** Thoracentesis pleural fluid analysis demonstrated exudative fluid LDH: lactate dehydrogenase

Investigations	Results	Reference range
Total serum protein	0.2 g/dl	6 to 8 g/dl
Pleural fluid protein	0.9 g/dl	<1.5 g/dl
Serum LDH	264 U/L	140-280 U/L
Pleural fluid LDH	94 U/L	N/A

Management and outcome

Per the oncology team's recommendation, a CT-guided biopsy was conducted on the liver mass. Subsequent examination of the pathology report revealed the presence of a low-grade NET characterized by an acinar and festoon-like arrangement of cells. The tumor cells tested positive for synaptophysin and CD56, while KI-67 immunostaining indicated a proliferation index ranging from 1% to 2%. This comprehensive analysis led to the diagnosis of stage IV metastatic low-grade NET originating from the small bowel.

Significantly, given elevated levels of serum serotonin, positive chromogranin A, and evidence of right-sided valvular involvement (severe tricuspid regurgitation) as observed in the echocardiogram, coupled with the stage IV metastatic low-grade NET, a conclusive diagnosis of carcinoid heart disease was established.

The patient was discharged with a follow-up appointment with oncology. A discussion about the prognosis was held with the patient and his family, and they opted to pursue treatment. A dotatate scan was scheduled before starting treatment; however, the patient suffered cardiac arrest before the scan was conducted. He was brought to the hospital, where a poor prognosis was confirmed. After consulting with his family, the decision was made to focus on comfort measures, and the patient sadly passed away shortly thereafter.

This amalgamation of diagnostic evidence underscores the intricate nature of the patient's condition, consolidating the understanding of the underlying pathophysiology.

## Discussion

Carcinoid tumors have hemodynamic and structural implications for the entire body. The most frequent sites for carcinoid tumors are the gastrointestinal (GI) tract (73.7%) and the bronchopulmonary system (25.1%). Within the GI tract, most occur in the small bowel (28.7%), appendix (18.9%), and rectum (12.6%) [4]. Characteristically, when there is cardiac involvement, like the patient presented in this case study, it presents with thickening of the right side of the heart and structural valvular lesions leading to right-sided heart failure [5]. Higher concentrations of 5-hydroxyindoleacetic acid (5-HIAA) in patients with carcinoid cardiac disease as compared to patients with carcinoid disease with no heart involvement suggest that serotonin exposure to the right side of the heart leads to the development of cardiac carcinoid plaques [6-7]. However, much is still to be studied around carcinoid tumors, and the etiology is not completely understood given that cardiac lesions still occur despite aggressive treatment and control of serotonin release [8].

Carcinoid tumors, much like other malignancies, progress rapidly and present a challenging prognosis. Timely diagnosis and effective management are paramount to mitigating potential complications associated with this type of tumor. Notably, it may manifest as carcinoid heart disease, a complication arising from the excessive release of vasoactive amines into the systemic circulation. This abnormal release contributes to valvular abnormalities and heart failure, emphasizing the critical importance of swift intervention to address this specific complication and improve overall patient outcomes. Studies have shown that there may be a correlation between the severity of tricuspid valve regurgitation and prognosis. Advanced cardiac heart failure and right ventricular size are considered key factors in predicting outcomes of carcinoid disease because the hemodynamic ramifications contribute to high mortality [9]. Treatment for carcinoid cardiac disease includes somatostatin analogs, liver dearterialization, and cytotoxic chemotherapy, which has not been very promising in regard to survival rates [10]. However, intervention with cardiac surgery in the form of tricuspid valve replacement improved functional status and encouraged right ventricular remodeling [11].

Owing to the rarity of the disease, few studies have investigated patients with carcinoid heart disease, making this case study not only important but also a contribution to the overall research. It highlights the importance of recognizing carcinoid syndrome and the need for timely diagnosis and management. With appropriate treatment, patients with carcinoid syndrome can experience symptom relief and improved outcomes.

## Conclusions

This case report sheds light on the intricate interplay between NETs and carcinoid heart syndrome (CHS), presenting a rare but critical clinical scenario. Through meticulous presentation and management, we elucidate the challenges in diagnosing and treating patients with metastatic NETs and associated cardiac complications. This case underscores the necessity of a multidisciplinary approach involving oncology, cardiology, and other specialties to optimize patient care and outcomes. Furthermore, it highlights the ongoing need for research to deepen our understanding of the pathophysiology of CHS and to develop more effective diagnostic and therapeutic strategies for patients facing this complex condition. By sharing this case, we contribute to the collective knowledge base, fostering advancements in the management of NETs and associated cardiac manifestations, ultimately striving to improve the quality of care and prognosis for affected individuals.
